# Differentiating benign and malignant thyroid nodules: A cross‐sectional study on the comparison of diagnostic value of ultrasound elastography and fine needle aspiration biopsy

**DOI:** 10.1002/hsr2.1619

**Published:** 2023-10-10

**Authors:** Farnood Rajabzadeh, Ehsan Hassannejad, Iman Akhlaghipour, Mohammad Javad Imen, Atefeh Babazadeh Baghan, Ladan Goshayeshi, Seyed Morteza Taghavi, Shohreh Vojouhi, Asma Payandeh, AmirAli Moodi Ghalibaf

**Affiliations:** ^1^ Department of Radiology, Faculty of Medicine, Mashhad Medical Sciences Islamic Azad University Mashhad Iran; ^2^ Department of Radiology, School of Medicine Birjand University of Medical Sciences Birjand Iran; ^3^ Student Research Committee, Faculty of Medicine Mashhad University of Medical Sciences Mashhad Iran; ^4^ Mashhad Medical Sciences Islamic Azad University Mashhad Iran; ^5^ Department of Gastroentrology and Hepatology, Faculty of Medicine Mashhad University of Medical Sciences Mashhad Iran; ^6^ Surgical Oncology Research Center, Imam Reza Hospital, Faculty of Medicine Mashhad University of Medical Sciences Mashhad Iran; ^7^ Endocrine Research Center, School of Medicine Mashhad University of Medical Sciences Mashhad Iran; ^8^ Faculty of Medicine Mashhad University of Medical Sciences Mashhad Iran; ^9^ Student Research Committee, Faculty of Medicine Birjand University of Medical Sciences Birjand Iran

**Keywords:** cancer, elastography, fine needle aspiration, thyroid nodules

## Abstract

**Background and Aim:**

This study examines the comparison of ultrasound elastography and fine needle aspiration (FNA) in diagnosing thyroid cancers and investigates the use of elastography as the initial diagnostic test of thyroid cancers to avoid the need for invasive diagnostic tests.

**Methods:**

In this study, 28 patients with 48 thyroid nodules (TNs) who were candidates for FNA or surgery were examined within a period of 18 months. Cut‐off and subsequently sensitivity and specificity for elastography results, compared to pathology results as the gold standard, were calculated using the receiver operating characteristic curve (ROC).

**Results:**

Based on ROC, the cut‐off point differentiating the tissue stiffness between benign and malignant TNs was 25.400 kilopascal (kPa) (sensitivity of 90.9% and specificity of 78.4%). It was observed that age affects the tissue stiffness; therefore, the cut‐off was defined as 65.625 kpa for age groups under 50 years old (sensitivity of 100% and specificity of 100%) and 25.400 kpa for the age group above 50 years old (sensitivity of 88.9% and specificity of 70.4%).

**Conclusion:**

Based on the high sensitivity and specificity of shear wave elastography in the differentiation of benign and malignant TNs, it can be employed as a stand‐alone or in combination with other diagnostic techniques to reduce the need for inessential surgical operations. However, future studies or developments are needed on this promising diagnostic technique.

## 1 BACKGROUND

As the most common malignant tumors of the endocrine system, thyroid cancer accounts for 1−2% of the total cancer diagnoses in the world and has become more prevalent in the past decade. Therefore, it is absolutely critical to diagnose thyroid cancer as early as possible and improve the ability to differentiate between benign and malignant thyroid nodules (TNs).^1^ Palpable TNs are seen in roughly 5% of adults, but the prevalence may vary in different parts of the world. This number is typically higher in regions with iodine deficiency and with a population of older people (especially older women). Due to the high prevalence, physicians usually encounter TNs in physical examinations or in accidental findings while conducting medical imaging for different purposes.^2^, ^3^, ^4^ The size of the majority of TNs is more than 1 cm; however, the clinical diagnosis depends on the nodule location, the anatomy of the patient's neck, and the experience and competence of the examiner. More sensitive diagnostic techniques such as CT‐Scan, thyroid ultrasounds, and pathologic examinations detect TNs in 50% of adults above 50 years old. This high number of random encounters with thyroid lesions highlights the need for more discussions on how TNs are diagnosed and what nodules may require additional evaluation.^2^

Around 95% of TNs are benign. Fine‐needle aspiration (FNA) and cytology are conducted to identify the limited malignant group of suspicious nodules discovered based on TSH, radionuclide scan, and ultrasound examinations. Based on some major studies, FNA biopsies showed the following results: 65% benign, 5% malignant or suspected malignant, 10% non‐diagnosed or insufficient signs to diagnose, and 20% known. In an ideal situation, if an expert cytopathologist performs ultrasound‐guided FNA, it possesses pretty high sensitivity and specificity.^5^, ^6^ Since most thyroid cancers are “hard,” evaluation of the tissue stiffness has become one of the significant characteristics of TNs.^4^ Elastography is a noninvasive medical imaging technique demonstrating soft tissue's elastic characteristics and stiffness. Since the thyroid is an accessible organ and elastography results are immediate, it is widely considered a suitable tool to evaluate TNs.^7^, ^8^

Studies have shown that the sensitivity and specificity of elastography in differentiating benign and malignant TNs are superior compared to traditional techniques.^7^ Many studies have shown encouraging results for the use of elastography, and implementation of this technique has the potential to reduce the number of inessential surgical operations on benign TNs.^7^, ^9^, ^10^, ^11^, ^12^, ^13^ The aim of this study is to compare the FNA and elastography in diagnosing the types of TNs as two invasive and noninvasive techniques, respectively. The goal is to investigate whether elastography has the potential to replace the FNA as the invasive technique for the initial diagnosis of thyroid lesions.

## 2 METHODS

### 2.1 Study design

In a cross‐sectional study, patients diagnosed by an endocrinologist with inactive nodules who were candidates for FNAs (having higher‐than‐normal TSH, or with cold nodules visible in a radionuclide scan) or who were surgery candidates due to the results of FNA, ultrasound, or clinical signs, were selected for this research. Typically, all patients who were candidates for FNA initially went through an ultrasound evaluation to identify the nodule's size, precise location, and nature (cystic or solid). Additionally, elastography was completed using shear wave elastography (SWE) (Supersonic, Aixplorer, Linear Probe‐15‐4) to measure the stiffness of TNs. The Medical Ethics Committee of Mashhad Medical Science Islamic Azad University approved this study and all patients and their families were informed and signed the informed consent.

### 2.2 Procedures and outcomes

Patients were asked to hold their breath and not swallow for a short time while the image of the SWE was formed. The transducer was held in a stable position without performing any compression over the gland to minimize the artifacts resulting from compression (freehand technique), then SWE was started upon the conventional sonographic image. Quantitative elastography assessment was performed using a 2 × 2 mm region of interest (ROI), with the transducer positioned at the stiffest area, avoiding cystic areas, calcification, and normal thyroid tissue. All measurements were recorded and saved in kilopascal (kPa). Three measurements were taken for each nodule: the minimum, maximum, and mean SWE. The examination was repeated three times for each lesion, and the average of the three measurements was used as the final result. In case sampling was conducted by fine needle and the patient was a candidate for surgery, elastography was completed at least 15 days after the fine needle sampling and before the surgery to avoid any potential hematoma. Based on the sampling results, some patients were considered candidates for thyroid surgical operation while others were excluded from our study. The histopathology sample of patients going under surgery was used as the diagnostic indicator based on which nodules were categorized into two groups of “benign” and “malignant” types and were compared with measured stiffness in elastography. This made available a diagnostic level for differentiating benign nodules from malignant ones in elastography. Subsequently, the sensitivity and specificity of elastography were calculated in distinguishing benign and malignant TNs.

### 2.3 Statistical analysis

Tables and statistical indexes such as mean and median were used in describing data, and in data analysis, data preliminary passed the Mann−Whitney's *U* test for normality. The receiver operating characteristic (ROC) curve was used to obtain the cut‐off value. The software used in this study was SPSS v.25 and statistical significance was considered for a *p* Value less than 0.05 (*p* ≤ 0.05). *p* Values less than 0.05 are marked with “*” and the ones less than 0.01 are marked with “**” in the data presented.

## 3 RESULTS

This study was conducted within 18 months (September 2017 to February 2019) on 28 patients (21 women, i.e., 75% and 7 men, i.e., 25%) with an average age was 54.5 (22−77 years old range). Patients in the study included 48 TNs (some patients had more than 1 TN). They were candidates for thyroid surgery based on the results of sonographic findings and sampling by a fine needle.

Among the 12 patients with solitary TNs, eight patients were diagnosed with benign nodules after the surgery and based on the final histological results. The results of four patients came back showing malignant nodules, including three papillary carcinomas and one follicular carcinoma.

Among the 16 patients with multiple TNs (36 nodules), the histological results showed 12 patients (27 nodules) as benign. Biopsy results of three patients (six nodules) showed two papillary carcinomas and one follicular carcinoma. Histological results showed one patient (three nodules) with two benign nodules and one follicular carcinoma.

**Figure 1:**
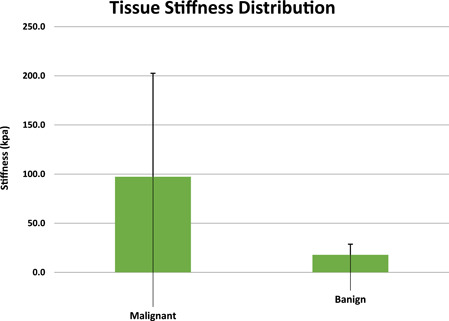
Mean tissue stiffness distribution based on pathology of thyroid nodules.

In mean tissue stiffness distribution analysis based on the pathology of TNs, the hypothesis of normality was rejected (*p* < 0.05) based on the Shapiro−Wilk test. The results showed a considerable and meaningful difference between the two groups on stiffness (Mann−Whitney's *U* test) with a *p*‐value equal to 0.0001 ** as results are listed in Figure 1.

The measured tissue stiffness by SWE for malignant nodules (mean of 97.4 kpa; range of 15.8–297.25 kpa) was significantly higher than stiffness for benign nodules (mean of 17.8 kpa; range of 4−38 kpa) (*p* Value at 0.0001**) (Table 1).

**Table 1:** Tissue stiffness distribution based on pathology of thyroid nodules.

Group	Min (Kpa)	Max (Kpa)	Median (Kpa)	Mean (Kpa)	Standard Deviation	Statistical Indexes
Malignant	15.8	297/25	31/50	97/40	105/15	*U* = −3.72
Benign	4	38	14/25	17/86	10/84	*p* = 0.0001**
Total	4	297/25	20/25	36/09	59/86	

** Significance level: *p* value <0.01

**Figure 2:**
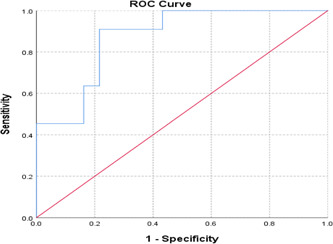
Cut‐off distribution for tissue stiffness.

As results are shown in Figure 2, Table 2, and Table 3, The tissue stiffness that can be used to differentiate benign TNs from malignant ones was calculated by the ROC curve to be 25.400 kpa (sensitivity of 90.9% and specificity of 78.4%) and a TN with tissue stiffness equal or greater than this value is most probably malignant.

**Table 2:** Area under the ROC curve.

**Asymptotic 95% confidence interval**		**Asymptotic Sig.** ^b^	**Std. error** ^a^	**Area under RCI**
**Upper bound**	**Lower bound**			
0/978	0/0767	0/000	0/054	0/872

*Note*: Reliable change index, RCI > 1.96 considered significant.

Abbreviation: ROC, receiver operating characteristic.

^a^ Under the nonparametric assumption;

^b^ Null hypothesis: true area = 0.5.

**Table 3:** ROC cut‐off values.

Specificity	Sensitivity	Positive if greater than or equal to
0.216	0.909	25.400
0.216	0.818	26.275
0.216	0.636	27.250
0.189	0.636	28.875
0.162	0.636	30.750
0.162	0.455	33.850
0.135	0.455	36.450
0.108	0.455	36.800
0.081	0.455	36.950
0.027	0.455	37.500

**Figure 3:**
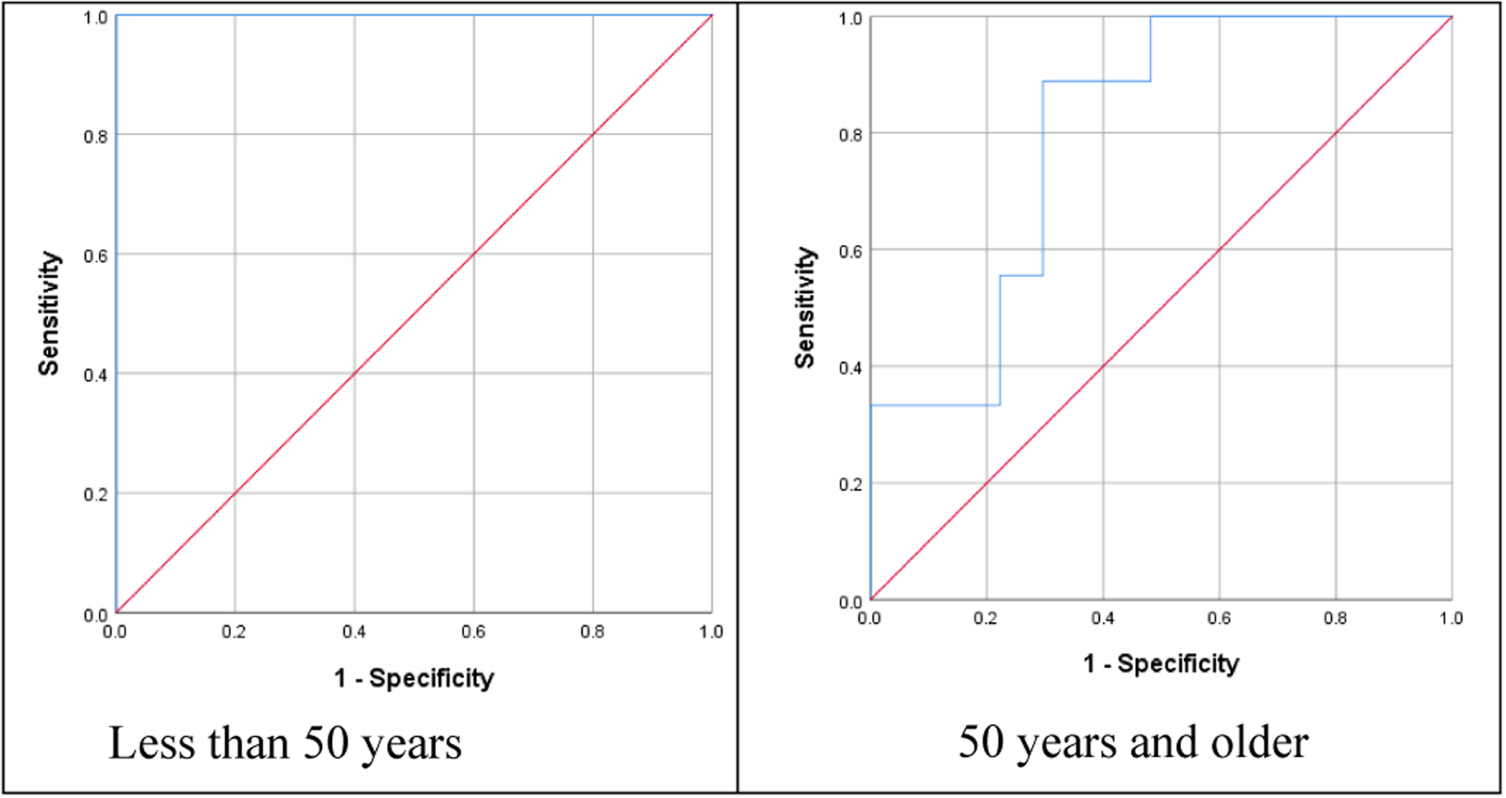
ROC curve based on age of the patients. ROC, receiver operating characteristic.

The patient's age makes a difference in measuring tissue stiffness if we categorize patients to under and above 50 years old, the difference is seen at the diagnostic level. (Figure 3 and Table 4).

**Table 4:** Area under ROC curve based on age of the patients.

**Asymptotic 95% confidence interval**		**Asymptotic Sig.** b	**Std. error** a	**Area**	**Age**
**Upper bound**	**Lower bound**				
1.000	1.000	0.032	0.000	1.000	>50
0.948	0.649	0.008	0.076	0.798	≤50

Abbreviation: ROC, receiver operating characteristic.

^a^ Under the nonparametric assumption;

^b^ Null hypothesis: true area = 0.5.

It was found that due to the significant differences in tissue stiffness in different ages, it is better to define a separate ROC cut‐off value for every age group to differentiate between the benign and malignant TNs. In this study, we divided the patients into two groups of younger and older than 50 years old and defined a ROC cut‐off value for each group separately. For the under 50 years old group, it was calculated as 65.625 kpa (sensitivity of 100% and specificity of 100%), and for the 50 years old and above, the value was calculated to be 25.400 kpa (sensitivity of 88.9% and specificity of 70.4%). (Table 5).

**Table 5:** ROC Curve cut‐off based on patient age.

Age	10Specificity	Sensitivity	Positive if greater than or equal to
Under 50 year old	0.200	1.000	18.950
	0.100	1.000	23.000
	0.000	1.000	65.625
	0.000	0.500	201.750
50 years old and above	0.370	0.889	23.200
	0.333	0.889	24.500
	0.296	0.889	25.400
	0.296	0.778	26.275

## 4 DISCUSSION

TNs are a common problem in most communities. While the majority of the TNs are without any symptoms and the chance of being malignant is 5−10%, the general prevalence of the issue depends on age, gender, history of receiving any radiation therapy, and the history of family thyroid cancer.^14^, ^15^ Guidelines based on observations of the American Thyroid Association and the American Association of Clinical Endocrinology recommend FNA sampling of TNs based on sonographic results.^2^ Since the malignant tissues have higher stiffness in the lesion location than the benign tissues, examining elasticity and tissue stiffness in its location using a hand is the oldest and most common technique to examine the TNs. Therefore, elastography is a noninvasive technique in ultrasound examinations based on which data can be obtained on stiffness.^10^ Ultrasound elastography can compare the difference in elastic coefficient between different tissues and determine tissue stiffness by measuring the amplitude of signal movement before and after compression.^16^ In an ideal situation, if ultrasound‐guided FNA is completed by an expert cytopathologist, it possesses quite high sensitivity and specificity to diagnose cancer.^5^ Quantitative elastography is a complex technique to determine tissue stiffness to review soft tissue's elasticity values and mechanical properties based on its constructive macromolecules' composition and structure. The ultrasound probe obtained the stiffness of the TN and normal tissue of thyroid parenchyma using pressure on the thyroid tissue location. Quantitative elastography makes it possible to draw the diagram of the elasticity of a ROI versus time, in consecutive periods of tissue pressure and resting. These diagrams can quantitatively evaluate the tissue stiffness value.^10^, ^16^, ^17^, ^18^, ^19^

In this study, the goal was to compare the diagnostic capabilities of elastography and the FNA sampling to determine the types of TNs suspected to be malignant. It was determined that measured tissue stiffness by SWE for malignant nodules (mean = 97.4 kpa) is significantly higher than stiffness for benign nodules (mean = 17.8 kpa) (*p* = 0.0001).

Furthermore, studying ROC curves showed that the cut‐off value of tissue stiffness to differentiate benign and malignant TNs was 25.400 kpa with a sensitivity of 90.9% and specificity of 78.4%, so any TNs with tissue stiffness higher than this number are most probably malignant.

The patient's age affected the difference in tissue stiffness measurements; thus, dividing patients into two groups under and above 50 years old made this difference more meaningful. The extracellular matrix in every part of the body consists of elastic fibers. Aging increases melanin elastic fibers but reduces the oxytalan elastic fibers, which results in changes in the quantity and morphology of elastic fibers and thus the tissue elasticity and stiffness.^20^, ^21^ Therefore, it seems more logical to define a separate cut‐off value for every age group to differentiate the benign and malignant TNs. In this study, we divided the group of patients into below and above 50 years old patients and defined a separate cut‐off for each group: Under 50 years old (cut‐off = 65.625 kpa; sensitivity = 100%, specificity = 100%), and 50‐year‐old and above (cut‐off = 25.400 kpa, sensitivity = 88.9%, specificity = 70.4%).

Veyrieres et al.^22^ declared in their research entitled “A threshold value in SWE to rule out malignant TNs: a reality?” discussed that the proper cut‐off measured as a result of SWE to differentiate benign and malignant TNs is 66 kpa (4.70 m/s) with a sensitivity of 80% and specificity of 90.5%. This study has been cited as a reliable source in several guidelines consistent with our obtained results for our group of patients under 50 years old. There has been no study on the impact of age on TN stiffness and further research on the subject is recommended. Another fact is that ultrasound elastography‐ as shear‐wave elastography‐ is a method used in the diagnosis of inflammatory conditions.^23^, ^24^ On the other hand, TNs, especially when malignant, produce a significant amount of inflammatory burden.^25^, ^26^ Therefore, higher values in the malignant nodules could be due to their higher inflammatory activities; however, the role of the older age should not be ignored. Additionally, this fact could be as guide for further studies to investigate the inflammatory markers in ultrasound elastography‐based diagnosis of malignant nodules

Our study has some limitations. The sample size is small; therefore, a verification of a larger group is necessary. A multicenter approach would help collect more data on a larger scale. Due to the unavailability of the histopathological results for the group of patients who were not candidates to go under surgery, those patients were excluded from our study, so It was not possible to examine the impact of sensitivity and specificity of FNA in differentiating benign and malignant TNs in this study which may have impacted the elastography diagnostic worth of this study.

## 5 CONCLUSIONS

Despite the need for more studies on the subject and for further evidence, the capability of elastography in combination with FNA in reducing inessential surgeries for benign TNs is noteworthy, and it is concluded that the results of elastography in evaluating nodules with unknown cytology or unknown nature is quite helpful.

## AUTHOR CONTRIBUTIONS

**Farnood Rajabzadeh**: Conceptualization; supervision; validation; writing—original draft; writing—review and editing. **Ehsan Hassannejad**: Conceptualization; writing—original draft; writing—review and editing. **Iman Akhlaghipour**: Data curation; writing—original draft. **Mohammad Javad Imen**: Data curation; writing—original draft. **Atefeh Babazadeh Baghan**: Writing—original draft. **Ladan Goshayeshi**: Conceptualization; project administration; supervision. **Seyed Morteza Taghavi**: Writing—original draft. **Shohreh Vojouhi**: Writing—original draft. **Asma Payandeh**: Writing—original draft. **AmirAli Moodi Ghalibaf**: Writing—original draft; Writing—review and editing.

## CONFLICT OF INTEREST STATEMENT

The authors declare no conflict of interest.

## ETHICS STATEMENT

The method was approved in terms of compliance with scientific and ethical standards. All methods were performed in line with the relevant guidelines and regulations. The Medical Ethics Committee of Mashhad Medical Science Islamic Azad University approved this study and all patients and their families were informed and signed the informed consent.

## TRANSPARENCY STATEMENT

The lead author Ehsan Hassan Nejad affirms that this manuscript is an honest, accurate, and transparent account of the study being reported; that no important aspects of the study have been omitted; and that any discrepancies from the study as planned (and, if relevant, registered) have been explained.

## Data Availability

The data sets created during the current study are not publicly accessible due to the possibility of compromising the privacy of individuals.
